# The effect of applying eye movement exercise on pain and sleep quality among children undergoing chemotherapy

**DOI:** 10.1186/s12912-026-04750-5

**Published:** 2026-05-20

**Authors:** Nagwa Ibrahim Eldemery, Reham Elsaeed Hashad, Heba Abubakr Salama, Hend Reda Ali Elkest, Emmillie Joy Mejia, Ayman Muhammad Kamel Senosy, Shereen Ahmed Elwasefy

**Affiliations:** 1 Faculty of Nursing, Mansoura University, Mansoura, Egypt; 2 Faculty of Nursing, Tanta university, Tanta, Egypt; 3 MAPUA University, Manila, 1204 Philippines; 4 Faculty of Nursing, Ain Shams University, Cairo, Egypt; 5 Faculty of Nursing, Galala University, Suez, Egypt

**Keywords:** A quasi-experimental study, Nursing interventions, Children, Eye movement exercise, Sleep quality

## Abstract

**Background:**

Nursing interventions that apply neutropenic precautions, provide medication, offer nutritional and psychological support, and address side effects such as pain, nausea, fatigue, and infection are beneficial for children undergoing chemotherapy. Pain and sleep difficulties are common in children receiving chemotherapy. Therefore, this study looks at eye movement exercises as a non-pharmacological means of enhancing their comfort and overall health.

**Aim:**

This study aimed to evaluate effect of applying eye movement exercise on pain and sleep quality among children undergoing chemotherapy.

**Subjects and methods:**

To accomplish this study’s goal, a quasi-experimental research approach was employed. The research was carried out at the Oncology Center affiliated with Mansoura University. A selective sample of fifty children receiving chemotherapy in the setting was included in the study. Three tools were employed to gather data: Tool I: A structured interviewing questionnaire consisting of two sections (Part 1: Child characteristics; Part 2: Children’s medical history); Tool II: Pain Rating Scale; Tool III: Pittsburgh Sleep Quality Index (PSQI).

**Results:**

The findings of the current study revealed that the implementation of eye movement exercises reduced the mean pain levels among children receiving chemotherapy and improved their sleep quality.

**Conclusion:**

The findings of the current study indicate that a structured eye movement exercise program may contribute to reducing pain levels and improving sleep quality among children receiving chemotherapy. As a simple, non-invasive, and low-cost intervention, eye movement exercises can be considered as a supportive nursing strategy to enhance patient comfort during chemotherapy. However, further research with larger sample sizes and controlled study designs is recommended to strengthen the evidence and confirm the effectiveness of this intervention in pediatric oncology settings.

**Clinical trial number:**

Not applicable.

## Introduction

Cancer remains one of the leading causes of death among teenagers and young adults. Survival rates have greatly improved due to advances in cancer treatments and supportive care, particularly in high-income countries, where they currently exceed 80% [1]. Chemotherapy is a widely used therapy and is essential for controlling metastatic malignancies. Although effective, chemotherapy has significant drawbacks, including a range of debilitating side effects such as fatigue, pain, nausea, mucositis, and emotional distress. Patients also often experience immune suppression and drug resistance [2].

Children receiving chemotherapy often experience significant physical and psychological side effects, with pain and poor sleep quality among the most distressing. Pain can result not only from cancer itself but also from repeated medical procedures and the adverse effects of chemotherapy [3]. Anxiety, treatment-related discomfort, and psychological stress are major contributors to sleep disturbances. A vicious cycle exists between these symptoms: pain disrupts sleep, and inadequate sleep increases pain sensitivity, reducing quality of life and slowing recovery [4].

Non-pharmacological strategies have received increasing attention in pediatric oncology due to the limitations and potential adverse effects of pharmacological treatments [5]. Among these, Eye Movement Exercises (EMEs), inspired by the bilateral stimulation component of Eye Movement Desensitization and Reprocessing (EMDR) therapy, have been explored as simple supportive techniques that may help reduce pain-related distress and improve sleep quality. These exercises involve visually tracking moving stimuli and may facilitate relaxation, attentional distraction, and emotional regulation. While EMDR has been linked to neurobiological changes in brain regions involved in emotion processing, such as the amygdala and anterior cingulate cortex, the exact mechanisms through which isolated eye movement exercises exert their effects remain uncertain and warrant further research [6].

Eye movement exercises are especially suitable for children undergoing cancer treatment because they are non-invasive, easy to perform, and require no special equipment. Improved immunity, better treatment responses, and greater emotional stability have all been linked to better sleep. Therefore, using simple behavioral techniques like EMEs to address pain and sleep disturbances in children with cancer can lead to significant improvements in overall health outcomes and quality of life [7].

Although eye movement exercises have shown promising results in adults with trauma-related conditions, research on their use in pediatric oncology remains limited. This study aims to investigate the effects of Eye Movement Exercises on pain and sleep quality in children undergoing chemotherapy, providing a promising step toward more comprehensive, patient-centered care [8]. Preliminary evidence suggests potential benefits, but further research is needed to confirm their effectiveness in pediatric cancer patients.

### Significance of the study

Research on simple, non-pharmacological approaches to address these challenges is limited, despite the high prevalence of chemotherapy-related side effects in children, including pain and sleep disturbances. In adults, Eye Movement Exercises (EMEs) have shown potential to improve sleep quality, reduce pain, and promote relaxation. Their use in pediatric oncology remains largely unexplored [9]. This knowledge gap highlights the importance of investigating EMEs as a supportive care approach for children undergoing chemotherapy [10]. This study aims to provide evidence for a safe, affordable, and accessible intervention that could enhance the overall well-being of pediatric cancer patients by assessing its effects on pain and sleep quality. The findings may also inform nursing practice and support the integration of non-invasive interventions into pediatric cancer care plans.

### Aim of the study

This study aimed to evaluate the effect of applying eye movement exercise on pain and sleep quality among children undergoing chemotherapy.

### Research hypothesis

#### H1

There is a statistically significant difference in the mean pain intensity scores among children undergoing chemotherapy before, after one week and after one month of applying eye movement exercise.

#### H2

There is a statistically significant difference in the mean sleep quality scores among children undergoing chemotherapy before, after one week and after one month of applying eye movement exercise.

## Subjects and method

### Research design

A quasi-experimental one-group pre test–post test design was used to achieve the aim of this study.

### Sample

A purposive sample of 50 children who underwent chemotherapy in the above-mentioned setting, available from the beginning of October 2024 to the end of December 2024, aged 6–12 years, of both sexes, and willing to participate in the study, was included as the subjects. Children who were clinically unstable, receiving analgesic drugs before bedtime, or who refused to participate in the study were excluded.

### Sample size justification

The sample size was calculated using G*Power version 3.1.9.7. The calculation assumed an effect size of **d = 0.70**, representing a moderate-to-large effect according to Cohen’s guidelines (0.2 = small, 0.5 = medium, 0.8 = large), with a significance level (**α = 0.05**) and statistical power of **0.95**. The estimated minimum sample size was 45 participants. To account for possible dropouts or missing data, 10% was added, resulting in a final sample size of 50 children. The effect size assumption was based on previously reported moderate-to-large effects in related clinical studies, as well as on the anticipated clinically meaningful difference between groups.

### Setting

The study was conducted at the Oncology Center affiliated with Mansoura University.

### Tools of data collection

Three tools were utilized to collect the required data for this study.

#### Tool I: Structured interview questionnaire

This tool was developed by the researchers into Arabic language after an extensive review of relevant literature and clinical documentation. It comprises two parts:

- **Part 1**: Demographic characteristics of the child, including age, gender, birth order, educational level, and place of residence.
- **Part 2**: Medical history designed based on literature review and hospital records. It collects information regarding the child’s medical diagnosis, presenting clinical symptoms, and details related to chemotherapy treatment.

#### Tool II: Pain assessment scales

This tool consists of two subscales to evaluate pain intensity and behavioral response:

- Part 1: Wong-Baker FACES Pain Rating Scale.

Originally, it was developed by [11], this scale assesses self-reported pain in children using six illustrated facial expressions ranging from “no hurt” (score 0) to “hurts worst” (score 10). Pain intensity was categorized into mild (1–3), moderate (4–6), and severe (7–10) levels.

- Part 2: Behavioral Pain Rating Scale.

It was Adapted from [12] this scale evaluates observable pain-related behaviors, including sobbing, restlessness, physical movement, and verbal expressions. Each behavior is scored from 0 to 2, with the total score ranging from 0 to 10. Pain intensity was interpreted as minimal (scores 1–3) or absent (score 0), and the mean score was calculated accordingly.

#### Tool III: Pittsburgh sleep quality index (PSQI)

Sleep quality was assessed using the PSQI, developed by [13] and culturally adapted and validated for Arabic-speaking populations [14] to measure sleep quality and disturbances over the previous month. the PSQI (Arabic version) was administered via structured proxy-interviews with parents/caregivers alongside the children to ensure the accuracy of sleep quality data, The PSQI contains 24 items, of which 18 items contribute to scoring across seven components: subjective sleep quality, sleep latency, sleep duration, habitual sleep efficiency, sleep disturbances, use of sleeping medication, and daytime dysfunction. The remaining 6 items are descriptive and are used to collect additional information about sleep patterns but do not contribute to the numerical scoring. Each component is scored on a 0–3 scale, with higher scores indicating greater sleep difficulties. Component scores are summed to produce a global PSQI score ranging from 0 to 21. A global score of 5 or below indicates good sleep quality, whereas scores above 5 indicate poor sleep quality and can be further categorized as: 6–10 mild sleep problems, 11–15 moderate sleep problems, and 16–21 severe sleep problems.

### Operational definitions

#### Pain

In the present study, pain refers to self-reported moderate to severe pain with a score ≥ 4 as measured by the Pain Rating Scale [11].

#### Sleep quality

In the present study, sleep quality refers to self-reported sleep disturbances with a score from 0 to 21 based on the Pittsburgh Sleep Quality Index (PSQI). A global score of 5 or below indicates good sleep quality, whereas scores above 5 indicate poor sleep quality and can be further categorized as: 6–10 mild sleep problems, 11–15 moderate sleep problems, and 16–21 severe sleep problems [12].

#### Eye movement exercises

In the current study, eye movement exercises refer to the child’s ability to perform basic ocular movements, such as moving the eyes downward and inward (inferior rectus), upward and inward (superior rectus), upward and outward (inferior oblique), and downward and outward (superior oblique) [7].

### Validity and reliability of tools

Three experts, including professors of pediatric nursing from Mansoura University’s Faculty of Nursing and an oncology professor from the Faculty of Medicine, evaluated and revised the study tools’ content for validity, clarity, item sequence, and relevance or irrelevance. Every recommendation made by the panel was considered, and some of them received rewards for finishing the tools.

Reliability was assessed using Cronbach’s alpha, reported as 0.89 for the Pain Rating Scale, 0.82 for the sleep quality items, and 0.825 for the PSQI. The instruments were deemed reliable by the experts.

#### Pilot study

10% of the total study population (5 children who had received chemotherapy) took part in a pilot study to show the tools’ feasibility, clarity, and applicability as well as to gauge how long it would take to complete the study tools. The pilot study participants were not included in the study sample because the research instruments needed to be modified.

### II–Methods

#### Field work

**Fig. 1:**
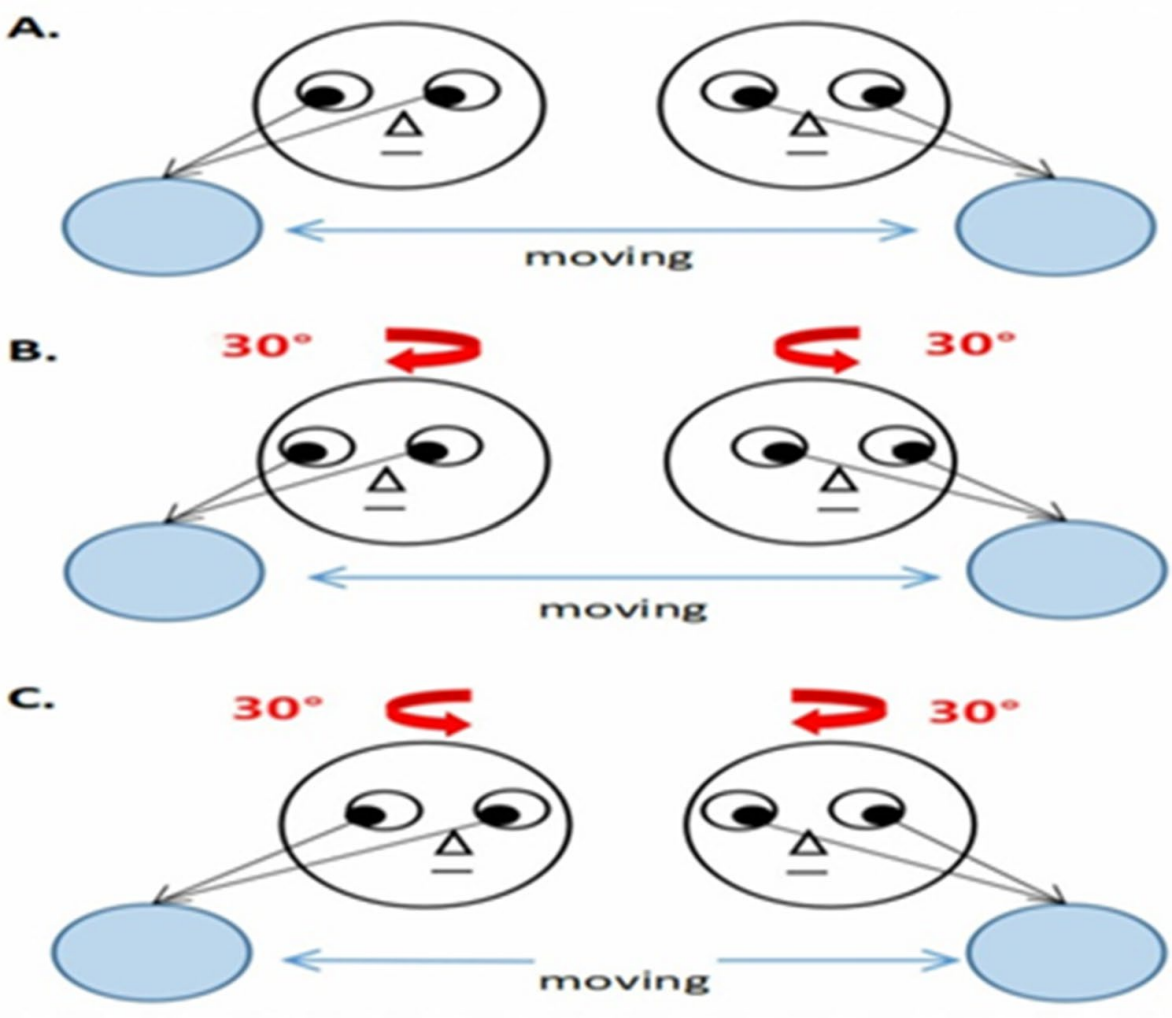
The finger-following eye movement exercise used during the intervention

- The university’s nursing faculty sent formal letters to the general director of the Mansoura University oncology center. Children and their parents give their verbal consent to participate in the study after being informed of its purpose.
- Data collecting for both sexes aged 6–12 years began in early October 2024 and ended in late December 2024.
- Three phases; assessment, implementation, and evaluation phases were used to collect data and to accomplish the current study’s aim: pre-intervention, after one week, and after one month.
- To secure oral agreement from both parents and children, the researchers conducted interviews with each kid to describe the objective, duration, and expected outcomes of the study. The researchers employed a range of instructional strategies, including group talks, demonstrations, and re-demonstrations, in addition to questions and discussions during the interview. A PowerPoint presentation along with several instructional resources.
- Standardized instruments, such as medical assessment sheets that included demographic and medical history information, including diagnosis, treatment details, and side effects of chemotherapy, were used to gather data twice a week (Tool I). Following the initial evaluation, the researchers used both subjective and objective pain rating measures (tool II) to observe pain for every kid undergoing chemotherapy. The researchers employed the Pittsburgh Sleep Quality Index (PSQI) (tool III) to evaluate the quality of each child’s sleep, and it took an average of 15 to 20 min to complete both tools.
- The researchers designed an intervention program based on eye movement exercises to improve pain perception and sleep quality among children undergoing chemotherapy. Participants were organized into ten groups, each including five children accompanied by their parents.
- The intervention program consisted of eight sessions conducted over four consecutive weeks (two sessions per week), including one theoretical and one practical session weekly, with each session lasting 30–45 min.
- The theoretical sessions aimed to introduce the concept of eye movement desensitization, explain its physiological and psychological effects on pain modulation and sleep regulation, and educate children (in age-appropriate language) and caregivers about the benefits of these techniques during cancer treatment. Visual aids and simple interactive materials were used to ensure understanding.
- The practical sessions involved guided eye movement exercises tailored to the child’s developmental stage. Children were taught horizontal and vertical eye tracking, blinking coordination with breathing, and focused gaze activities. The sessions included live demonstrations and supervised practice to ensure correct performance.
- Each practical session consisted of three phases:
- Preparation Phase (5 min): A brief assessment of the child’s current pain level followed by relaxation breathing exercises to ensure engagement and readiness for the activity.
- Bilateral Stimulation Phase (20 min): Using the finger-following technique, the researcher moved the index finger horizontally across the child’s visual field at 30–40 cm. The child was instructed to follow the finger movement using only the eyes while keeping the head stationary. The movements were repeated in sets of 20–30 passes, with short rest periods between sets to prevent ocular fatigue (see Fig. 1).
- Closure Phase (5 min): A brief verbal discussion with the child and caregiver, followed by reviewing the home-practice diary. To ensure correct performance, children were asked to re-demonstrate the technique under nursing supervision before continuing independent practice.
- The sample studied received eye movement exercises using instructional videos and written flyers. The researchers first explained the purpose of the eye movement training to the participants. Children were instructed to sit in a quiet and relaxed position while keeping their eyes focused horizontally on distance. The eyeballs were then moved in different directions, including inward (medial rectus), upward–inward (superior rectus), downward–inward (inferior rectus), upward–outward (inferior oblique), and downward–outward (superior oblique). Each movement was repeated 36 times, followed by circular eye movements from left to right to the maximum comfortable range. Each session lasted approximately 15–30 min. After the demonstration, participants were asked to re-demonstrate the exercise to ensure correct performance. Children were instructed to practice exercises once daily before bedtime for four weeks.
- Monitoring Home Practice: To ensure the accuracy of the Eye Movement Exercise (EME) performed at home, parental supervision and training: At least one parent/caregiver was trained alongside the child. Parents were instructed to act as “co-facilitators” at home, providing the external stimulus (finger movement) to ensure the child maintained the correct focal distance (30–40 cm) and rhythmic pace.
- Home Exercise Diary: A structured “EME Logbook” was provided to each family. Parents recorded the date, time, and duration of each session, along with a qualitative “Ease of Use” rating. These diaries were reviewed by the nursing team during the 1-week and 1-month follow-up assessments to calculate the adherence rate and address any technical difficulties.
- Using tools (I, II, III), each child was reassessed after the intervention and the effect of applying eye movement exercise on pain and sleep quality among children undergoing chemotherapy was evaluated after one week and after one month by using tool II and tool III and by comparing pre- and post-test results to determine its impact on pain reduction and improved sleep quality among the children. Furthermore, the researchers created a booklet with illustrations and verified content, which was given to the children under study as a self-learning aid. A pamphlet was created and given to them by the end of sessions.

### Statistical analysis

Statistical analysis was performed using SPSS version 20. Descriptive statistics were used to summarize the socio-demographic and clinical characteristics of the studied children, including frequencies, percentages, means, and standard deviations. Inferential statistics were applied to test the study hypotheses. Differences between categorical variables were examined using the Chi-square test. For continuous variables measured at three time points (before the intervention, after one week, and after one month), Repeated-Measures ANOVA was conducted to evaluate within-subject changes over time.

Prior to conducting ANOVA, normality of the residuals was assessed using the Shapiro–Wilk test, confirming that all continuous variables were approximately normally distributed (*p* > 0.05). The assumption of sphericity was tested using Mauchly’s test, and Greenhouse–Geisser corrections were applied when sphericity was violated. Post-hoc pairwise comparisons were performed using the Bonferroni adjustment to control multiple comparisons.

Effect sizes were calculated using partial eta squared (η²p) to quantify the magnitude of the intervention effect. According to Cohen’s guidelines, values of 0.01, 0.06, and ≥ 0.14 correspond to small, moderate, and large effects, respectively. A p-value < 0.05 was considered statistically significant, while *p* < 0.001 indicated a highly significant difference.

## Results

**Table 1:** The studied children’s distribution concerning their demographic characteristics (*n* = 50)

Socio-demographic data	No= 50	%
**Age in years**		
Six < eight years	30	58.0
Eight < ten years	18	38.0
Ten -Twelve years	2	4.0
**Mean ± SD 1.78 ± 7.88**		
**Gender**		
Boys	30	60.0
Girls	20	40.0
**Educational level**		
Primary school	48	96.0
Secondary school	2	4.0
**Residence**		
Rural	28	56.0
Urban	22	44.0

Table 1 illustrates that more than half (58%) of children were in the age group of 6 to 8 years, with a mean age of 1.78 ± 7.88 and 60% of them were boys. The majority (96%) of the children were in primary school period of education. Moreover, more than half (56%) of them reside in rural areas.

**Fig. 2:**
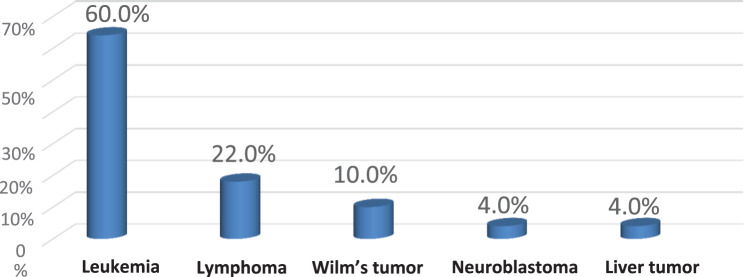
Children undergoing chemotherapy distribution regarding the medical diagnosis (*n* = 50)

The data shown in Fig. 2 indicates that three fifths of the children under study (60%) had a leukemia diagnosis, whereas 22% had a lymphoma diagnosis.

**Table 2:** Comparison between the studied children regarding their pain assessment using the Behavioral Rating scale before, after one week, and after one month of applying eye movement exercise. (*n* = 50)

Children’s responses to pain		Before applying eye movement exercise		After one week of applying eye movement exercise		*X*_*1*_*2* P value	After one month of applying eye movement exercise		*X*_*2*_*2* P value
		No.	%	No.	%		No.	%	
Crying	• Not crying	6	12.0	27	54.0	19.45 P = < 0.001 **	27	54.0	1.183 P = < 0.001 **
	• Crying but the response to Tender Loving Care (TLC).	9	18.0	13	26.0		11	22.0	
	• Crying and doesn’t respond to TLC	35	70.0	10	20.0		12	24.0	
Moving limbs during chemotherapy	• Nonmovement	0	0.0	27	54.0	16.55 P = < 0.001 **	33	66.0	1.654 P = < 0.001 **.
	• Partially bent	12	24.0	10	20.0		14	28.0	
	• Fully bent with finger flexion	38	76.0	13	26.0		3	6.0	
Agitation	• Child calm	0	0.0	24	48.0	25.99 P = < 0.001 **	32	64.0	2.347 P = < 0.001 **
	• Can be comforted to Lessen a gitation (mild).	15	30.0	14	28.0		12	24.0	
	• Can not be comforted.	35	70.0	12	24.0		6	12.0	

*Statistically significant at (*p* < 0.05) **Highly statistically significant at(*P* < 0.001)

*X*_*1*_^2^=chi-square Before vs. After one week, X_*2*_^2^=chi-square After one week vs. After one month

Table 2 shows the comparison of children’s behavioral responses to pain before the intervention, after one week, and after one month of applying eye movement exercises. Before the intervention, 70% of the children cried without responding to Tender Loving Care (TLC), 76% of them exhibited full limb flexion during chemotherapy, and 70% of them could not be comforted (70%). Following the intervention, a noticeable improvement in pain-related behaviors was observed. After one week and one month of eye movement exercises, the proportion of children who were calm, not crying, and showing no limb movement increased markedly to (64.0%, 54.0%, and 66.0% of the children, respectively) Cochran’s Q test revealed highly statistically significant differences across the three assessment periods (*p* < 0.001), indicating that the intervention was associated with a significant reduction in observable pain behaviors among the studied children.

**Fig. 3:**
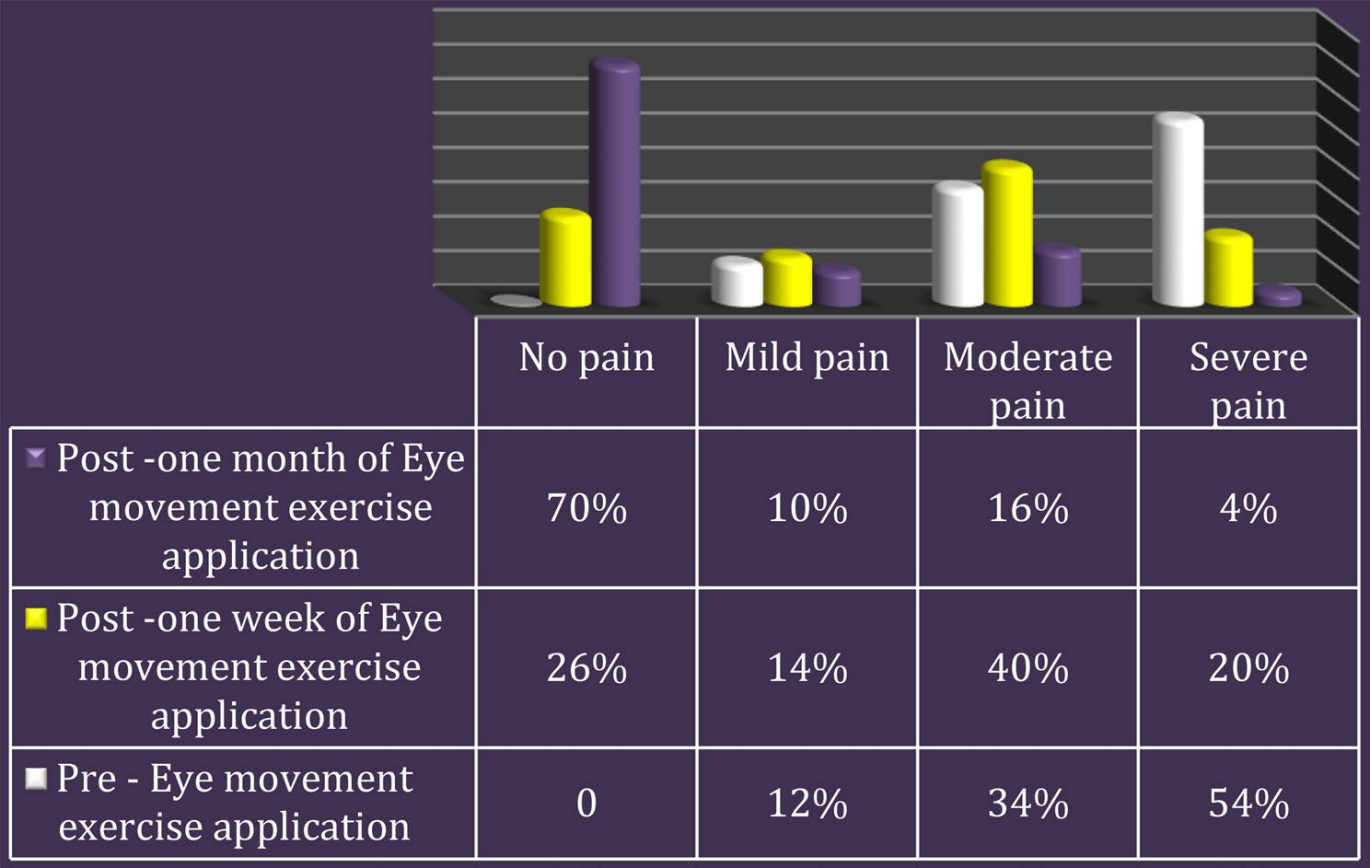
Total pain level among the studied children using the Wong-Baker faces pain rating scale before, after one week, and after one month of applying eye movement exercise. (*n* = 50)

Figure 3: After using the eye movement exercise, the children’s overall pain level decreased, The results revealed that before beginning the eye movement exercise, over half (54.0%) of the children in the study had excruciating discomfort; after a week, this number decreased to 20%. After using the eye movement exercise for a month, 70.0% of the children in the study reported no pain, while just 4.0% reported severe pain.

**Table 3:** Comparison between the Pittsburgh Sleep Quality Index mean scores among the studied children before, after one week, and after one month of applying eye movement exercise. (*n* = 50)

Sleep Quality	Before applying eye movement exercise		After one week of applying eye movement exercise		After one month of applying eye movement exercise	
	No.	%	No.	%	No.	%
♣ Good sleep quality (0–5)	--	--	--	--	--	--
♣ Mild sleep problem (6–10)	--	--	6	12.0	21	42.0
♣ Moderate sleep problem (11–15)	14	28.0	40	80.0	26	52.0
♣ Severe Sleep problems (16 to 21)	36	72.0	4	8.0	3	6.0
**Mean ± SD**	17.22 ± 1.65		12.42 ± 2.06		10.77 ± 2.26	
F- test	69.41					
**P-value**	< 0.001**					
**Effect size (Partial η²)**	0.58					

**Highly statistically significant at (*p* < 0.001. F: Repeated-Measures ANOVA, η²p: **Effect size (Partial η²)**

According to Table 3, the results indicate a progressive improvement in sleep quality over time. The mean PSQI score decreased from 17.22 ± 1.65 at baseline to 12.42 ± 2.06 after one week and further declined to 10.77 ± 2.26 after one month. Repeated-measures ANOVA showed a highly statistically significant difference across the three measurement points (F = 69.41, *p* < 0.001), with a large effect size (η²*p* = 0.58).

**Table 4:** Comparison between Pittsburgh Sleep Quality Index domains mean scores among the children studied before, after one week, and after one month of applying eye movement exercise. (*n* = 50)

Pittsburgh Sleep Quality Index domains	Before applying eye movement exercise	After one week of applying eye movement exercise	After one month of applying eye movement exercise	F Pvalue	Effect size (η²*p*)
Sleep latency	2.73 ± 0.44	2.43 ± 0.56	2.30 ± 0.70	8.912 0.002**	0.16
Subjective sleep quality	2.56 ± 0.50	1.03 ± 0.69	1.36 ± 0.85	35.279 <0.001**	0.42
Sleep duration	2.76 ± 0.43	2.06 ± 0.94	1.73 ± 1.14	21.147 < 0.001**	0.30
Sleep efficiency	2.96 ± 0.18	2.33 ± 1.12	1.73 ± 1.28	18.373 < 0.001**	0.27
Use of sleep medication	2.96 ± 0.18	2.13 ± 0.62	1.66 ± 0.71	9.542 0.003**	0.17
Daytime dysfunction	1.30 ± 0.83	0.96 ± 0.66	0.76 ± 0.62	16.824 < 0.001**	0.25
Sleep disturbance	1.86 ± 0.50	1.46 ± 0.50	1.23 ± 0.43	17.912 < 0.001**	0.23

**Highly statistically significant at (*P* < 0.001), F: Repeated-Measures ANOVA,

η²p: Partial Eta Squared to masur the effect size

Table 4 Clarifies that the mean PSQI domain scores among the studied children (*n* = 50) decreased significantly after one week and one month of applying eye movement exercises. Before the intervention, children had higher scores in sleep latency (2.73 ± 0.44), subjective sleep quality (2.56 ± 0.50), sleep duration (2.76 ± 0.43), and sleep efficiency (2.96 ± 0.18), indicating poor sleep quality. Following the intervention, scores declined across all domains, including use of sleep medication, daytime dysfunction, and sleep disturbances. Repeated-measures ANOVA revealed statistically significant improvements (*P* < 0.001), with large effect sizes in subjective sleep quality (η²*p* = 0.42), sleep duration (η²*p* = 0.30), and sleep efficiency (η²*p* = 0.27), indicating that eye movement exercises had a strong and clinically meaningful impact on the children’s sleep quality.

**Table 5:** Total Pittsburgh Sleep Quality Index mean scores among the children studied before, after one week, and after one month of applying eye movement exercise(*n* = 50)

Items	Before applying eye movement exercise	After one week of applying eye movement exercise	After one month of applying eye movement exercise
Mean ± SD	17.22 ± 1.83	13.42 ± 2.06	10.67 ± 2.77
F	68.53		
P value	< 0.001**		
Effect size (η²p)	0.59		

**Statistically significant at *p* < 0.001, F: Repeated-Measures ANOVA

η²p: Partial Eta Squared to masur the effect size

Table 5 indicates the total Pittsburgh Sleep Quality Index (PSQI) scores among the children studied before, after one week, and after one month of applying eye movement exercises. The findings show a marked reduction in the total PSQI scores following the intervention. Before applying eye movement exercises, the mean score was 17.22 ± 1.83, which decreased significantly to 13.42 ± 2.06 after one week, and further declined to 10.67 ± 2.77 after one month. Repeated-measures ANOVA indicated a highly statistically significant difference among the three measurements (F = 68.53, *p* < 0.001), with a large effect size (η²*p* = 0.59). These findings suggest that eye movement exercises were associated with significant improvements in overall sleep quality among the studied children.

## Discussion

Children receiving chemotherapy frequently experience pain and sleep disturbances, negatively affecting recovery and overall health. Eye movement exercises (EMEs) are a non-pharmacological intervention proposed to reduce pain and improve sleep quality. This study aimed to assess the effects of EMEs on pain and sleep in children undergoing chemotherapy [16].

In terms of the characteristics of the children under chemotherapy, the current study discovered that over half of the children were male and that the majority of the children were between the ages of 6 and 8. The findings of the current study are consistent with those of [17], who investigated " Childhood and adolescent cancer statistics” " and indicated that leukemia is the most common childhood cancer, particularly among early school-age children and with a slightly higher incidence in males. From the researcher’s point of view, this result may be explained by the fact that childhood cancer is more prevalent among males than females, while the predominance of 6–8-year-olds aligns with the higher incidence of cancers like leukemia in early school-age children.

Three fifths of the children of the youngsters in the research had leukemia, according to the study’s current findings. The current results are in line with those of a study conducted by [18], who examined “Cancer statistics.” and found that leukemia is the most common childhood malignancy, representing approximately one-third to nearly half of all pediatric cancer cases in recent population-based studies. The fact that leukemia is the most prevalent childhood cancer and necessitates frequent hospital stays and procedures, which raises its representation in pediatric oncology studies, may help to explain the current outcome.

The findings of the present study demonstrated an improvements in children’s responses to pain after the application of eye movement exercises. Crying, agitation, and limb movement were markedly reduced, with more than half of the children showing no crying or movement and remaining calm after one month. These current results are in harmony with [19] who studied " Non pharmacologic management of pain in pediatric oncology patients: A state of the science review” and mentioned that that supportive non-pharmacological interventions can reduce pain-related distress and improve coping behaviors in in the majority of children undergoing cancer treatment.

On the other hand, the findings of the present study are not in line with those of [20] who conducted study about " Effects of eye movement techniques on sleep quality and emotional regulation: A systematic review” and found that despite distraction techniques such as eye movement exercise, a considerable proportion of children continued to exhibit high levels of crying and agitation. The researchers rationalized the current findings in the light of fact that eye movement exercises may reduce children’s pain responses by stimulating brain regions responsible for sensory integration and emotional regulation, thus lowering the perception of pain. Psychologically, they function as an effective distraction technique that shifts focus away from painful stimuli, interrupts fear–distress cycles and gradually strengthens coping mechanisms over time.

Moreover, The findings of the present study demonstrated significant improvements in the total pain level of children after the application of eye movement exercises, in which, more than half of the studied children experienced severe pain before the intervention, which declined to lower percentage after one week while, after one month of application, the majority of the children reported no pain at all. These results are agreed with the findings of [21], who studied " Eye Movement Desensitization and Reprocessing (EMDR)*in pediatric hospital setting: A case report*” and reported that structured exercise interventions during chemotherapy not only improve physical fitness but also reduce pain intensity and enhance psychosocial well-being in most pediatric oncology patients. From the researchers’ point of view, the current results related to the fact that eye movement exercises may attenuate children’s pain by activating cortical and subcortical areas responsible for sensory integration and emotion regulation and hence, reducing the salience of pain signals.

The findings of the present study revealed a remarkable improvement in children’s sleep quality following the application of eye movement exercises as before the intervention, most of the studied children suffered from severe sleep problems which decreased to the vast minority of them after one week and further declined to 6% after one month of intervention. Furthermore, the mean Pittsburgh Sleep Quality Index (PSQI) mean scores also showed a progressive reduction, with highly significant differences observed across the three measurement points (F = 69.41, *p* < 0.001, η²*p* = 0.58), indicating a large effect size at **(***P* < 0.001). These results are consistent with [22], who conducted study about " Sleep patterns in children with cancer: Clinical implications for assessment and intervention " and demonstrated improvements in subjective sleep quality and overall sleep disturbances among children with cancer and most of the studied children shifted from severe sleep level to moderate levels, and severe cases dropped further to very low percentage after interventions. These current results may be due to those non-pharmacological interventions incorporating attentional distraction significantly improved sleep quality and reduced distress in children undergoing cancer treatment.

According to the current study’s findings, applying eye movement exercises has effectiveness on improving all Pittsburgh Sleep Quality Index (PSQI) domains. Higher mean scores for sleep latency, subjective sleep quality, duration, and efficiency before the intervention indicated that children had poor sleep patterns. But after a week, these scores sharply declined, and after a month, they continued to improve, suggesting improved sleep outcomes. Sleep latency and the use of sleep aids also showed substantial improvements (P < 0.05), and there were highly significant variations in the quality, duration, efficiency, daytime dysfunction, and disturbance of sleep (P < 0.001). These findings correspond with [23] who studied " non-pharmacological interventions for cancer-related insomnia: A systematic review and network meta-analysis based on Pittsburgh Sleep Quality Index (PSQI) and Insomnia Severity Index (ISI)” and highlighted that relaxation- and movement-based therapies can enhance sleep efficiency and reduce latency in children with chronic illness. Similarly [24], who conducted study about “non-pharmacological interventions for sleep disturbances in cancer patients: A scoping review” and confirmed that after two weeks of eye movement intervention, there were statistically significant differences in sleep efficiency, use of sleep medication, and sleep disturbance at (P < 0.001). The researcher rationalizes these findings by suggesting that eye movement exercises may down regulate hyper arousal, stabilize circadian rhythms, and act as a psychological distraction, collectively contributing to improved sleep quality.

However, the results of the current study show that the application of eye movement exercises significantly reduced the overall PSQI mean scores among the children under study. The mean score gradually decreased from the high PSQI score prior to the intervention to a lower mean score after one week and one month, indicating a noticeable improvement in overall sleep quality. These findings are consistent with [25], who investigated how eye movement training affected the Pittsburgh Sleep Quality Index-based sleep quality of patients with advanced lung cancer. They found that the intervention group’s PSQI scores significantly improved, their negative emotions decreased, and their pain levels decreased. These current findings may be related to the idea that eye movement exercises can help children sleep better by lowering physiological tension and anxiety, which makes them feel more at ease and calm.

### Limitations of the study

Several limitations should be acknowledged. The study used a quasi-experimental, one-group pre-posttest design without a control group, limiting causal inference. It was conducted at a single center with a small sample size (*n* = 50), which may reduce generalizability. The follow-up period was only one month, so long-term sustainability is unknown. Another issue is the absence of a control group, risk of evaluator bias, and lack of control for confounding variables. The potential confounding variables such as use of analgesics, disease progression, and treatment variability may influence the outcomes. Analgesic use was not controlled, and maturation or adaptation effects related to ongoing treatment cannot be ruled out. PSQI relied on parental reporting for younger children, and the researchers both implemented the intervention and collected outcome data, with blinding not feasible, potentially introducing assessor bias. Finally, the study was not registered as a clinical trial. Future studies should consider multi-center designs, larger samples, longer follow-up, independent blinded assessors, and clinical trial registration. The effect size assumption was based on previously reported moderate-to-large effects in related clinical studies, as well as on the anticipated clinically meaningful difference between groups. And the assumed effect size may be overestimated and that larger confirmatory studies are needed.

## Conclusion

Based on the findings of the current study, the researchers concluded that applying eye movement exercise has a positive effect on reducing pain and improving sleep quality among children undergoing chemotherapy as evidenced by a decrease in total pain levels and in total mean PSQI scores among the studied children after eye movement exercise application.

### Recommendations

The study recommends eye movement exercises should be incorporated into care for children undergoing chemotherapy to reduce pain and improve sleep quality alongside other therapies. Further research with larger samples is recommended to strengthen evidence-based practice.

## Data Availability

The datasets used and/or analyzed during the current study are available from the corresponding author on reasonable request.

## Abbreviations

PSQI: Sleep Quality Index
EMEs: Eye Movement Exercises
EMDR: Eye Movement Desensitization and Reprocessing
